# Dengue Virus Type 3, South Pacific Islands, 2013

**DOI:** 10.3201/eid2006.131413

**Published:** 2014-06

**Authors:** Van-Mai Cao-Lormeau, Claudine Roche, Didier Musso, Henri-Pierre Mallet, Tenneth Dalipanda, Alfred Dofai, Francisco Nogareda, Eric J. Nilles, John Aaskov

**Affiliations:** Institut Louis Malardé, Papeete, Tahiti, French Polynesia (V.-M. Cao-Lormeau, C. Roche, D. Musso);; Direction de la Santé, Papeete (H.-P. Mallet); Ministry of Health and Medical Services, Honiara, Solomon Islands (T. Dalipanda);; National Referral Hospital, Honiara (A. Dofai); World Health Organization, Suva, Fiji (F. Nogareda, E.J. Nilles);; Queensland University of Technology, Brisbane, Queensland, Australia (J. Aaskov)

**Keywords:** dengue, dengue virus, dengue virus type 3, viruses, serotype, genotype, phylogenetics, South Pacific Islands, Solomon Islands, French Polynesia

## Abstract

After an 18-year absence, dengue virus serotype 3 reemerged in the South Pacific Islands in 2013. Outbreaks in western (Solomon Islands) and eastern (French Polynesia) regions were caused by different genotypes. This finding suggested that immunity against dengue virus serotype, rather than virus genotype, was the principal determinant of reemergence.

In contrast to circulation in countries in Southeast Asia, where dengue is hyperendemic and ≤4 dengue virus (DENV) serotypes might co-circulate, it is rare for >1 DENV serotype to sustainably circulate in any South Pacific island country or territory. The pattern of single-serotype predominance has been historically observed in the entire South Pacific region (Figure 1); 1 serotype circulates for 4–5 years before being displaced by another serotype, i.e., DENV-3 (1989–1996), DENV-2 (1996–2000), DENV-1 (2001–2009), and DENV-4 (2008–2009) (*1**–**5*). Because DENV-3 had not circulated in the South Pacific Islands since 1996, a large nonimmune human population susceptible to infection with this serotype was present, and it had been suggested that this serotype would reemerge in ≈2012 (*4*). 

**Figure 1:**
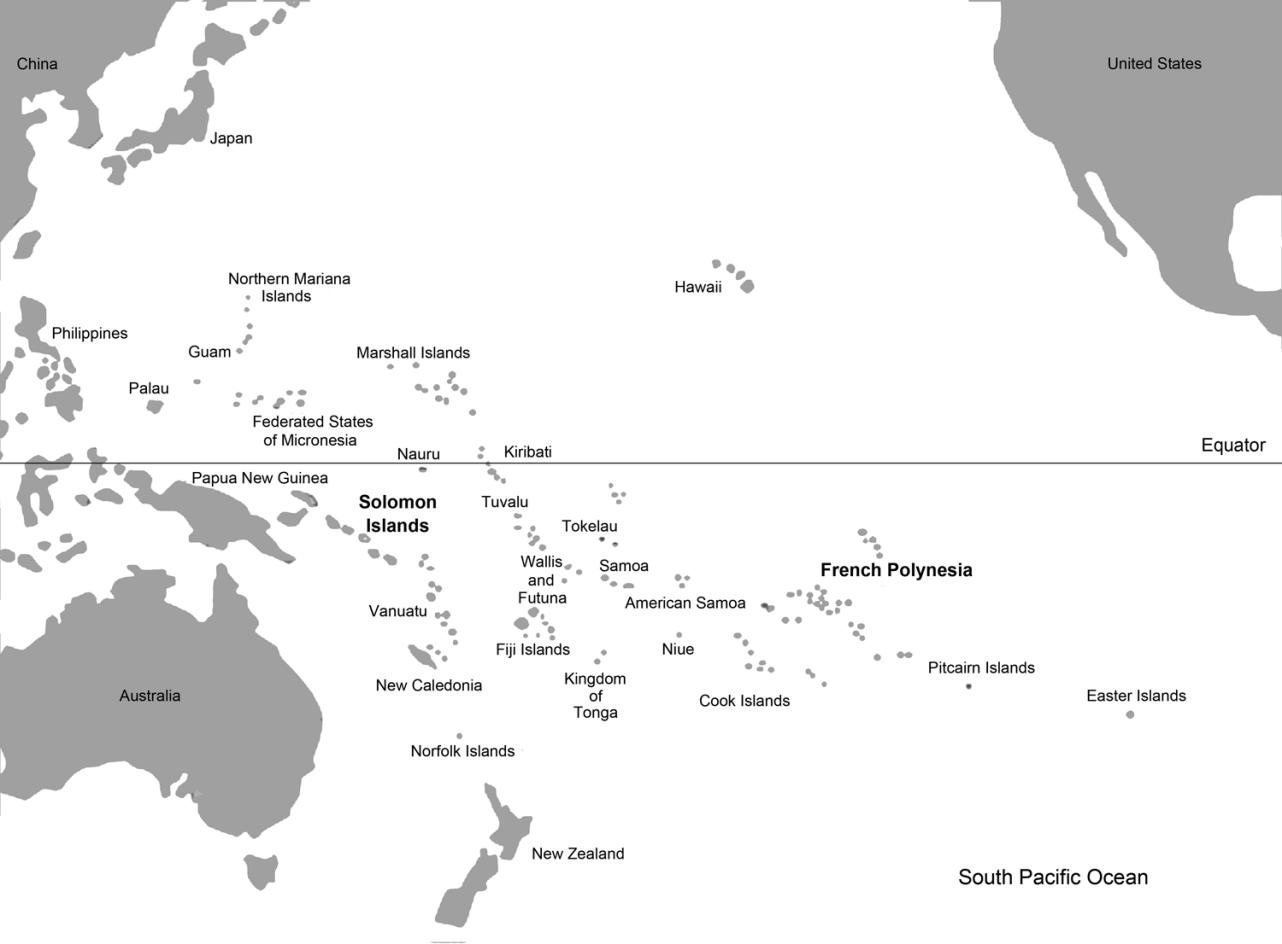
South Pacific Region showing the study areas (Solomon Islands and French Polynesia) tested for dengue virus type 3.

In January 2013, an outbreak of DENV-3 infections was reported in the Solomon Islands (*6*). Two months later, DENV-1 and DENV-3 infections were identified in patients in French Polynesia. DENV-3 isolated in the Solomon Islands belonged to genotype I, and DENV-3 isolated in French Polynesia belonged to genotype III. This finding is an example of dengue outbreaks in the South Pacific Islands caused by introductions of multiple genotypes of the same DENV serotype into susceptible populations in the South Pacific Islands (*2*), in this instance, genotype I from Southeast Asia and genotype III from South America.

## The Study

A dengue outbreak was reported by clinicians at the National Referral Hospital in Honiara, Solomon Islands, in January 2013. By July, >6,000 cases of suspected dengue had been reported, and 7 deaths were attributed to this outbreak. Serum samples from 3,141 patients were tested for DENV nonstructural protein 1 (NS1) and IgM against DENV (Dengue Duo, Standard Diagnostics Inc., Suwon, South Korea), and 1,220 (39%) samples were positive. DENV-3 was isolated by cell culture from 4 NS1-positive and IgM-negative samples by the World Health Organization Collaborating Centre for Arbovirus Reference and Research (Brisbane, Queensland, Australia). Ten additional NS1-positive samples were positive by reverse transcription PCR (RT-PCR) for DENV-3 when tested at the Institut Louis Malardé (Papeete, Tahiti, French Polynesia) (*6*).

 In the first week of March 2013, DENV-3 was detected in French Polynesia in 2 patients in the same family cluster, 1 of whom had returned with a fever from Cayenne, French Guiana, 2 weeks earlier. Patients infected with DENV-1 were also detected in French Polynesia in February 2013. By July 2013, a total of 1,326 suspected dengue cases had been reported, of which 258 were laboratory confirmed by NS1 ELISA (Bio-Rad, Hercules, CA, USA), IgM ELISA (Bio-Rad), or RT-PCR. Serotype identification confirmed 170 DENV-1 infections, 73 DENV-3 infections, and 1 co-infection with both virus serotypes.

Envelope genes from DENV-3 isolated in the Solomon Islands and French Polynesia were amplified by using RT-PCR and sequenced as described (*7**–**10*). The 2 sequenced DENV-3 isolates from the Solomon Islands were obtained in February 2013 from patients who lived in the capital (Honiara), where the outbreak occurred. The 5 sequenced DENV-3 isolates from French Polynesia were obtained at different times and distances from the initially detected case. One virus (PF13/120213) was obtained in February from the initial family cluster in Mahina, Tahiti; 2 viruses (PF13/280213 and PF13/260313) were obtained in February and March from patients who lived in Mahina; 1 virus (PF13/040313) was obtained in March from a patient who lived in Papeete, Tahiti; and 1 virus (PF13/230413) was obtained in April from a patient who lived on Moorea Island. 

**Figure 2:**
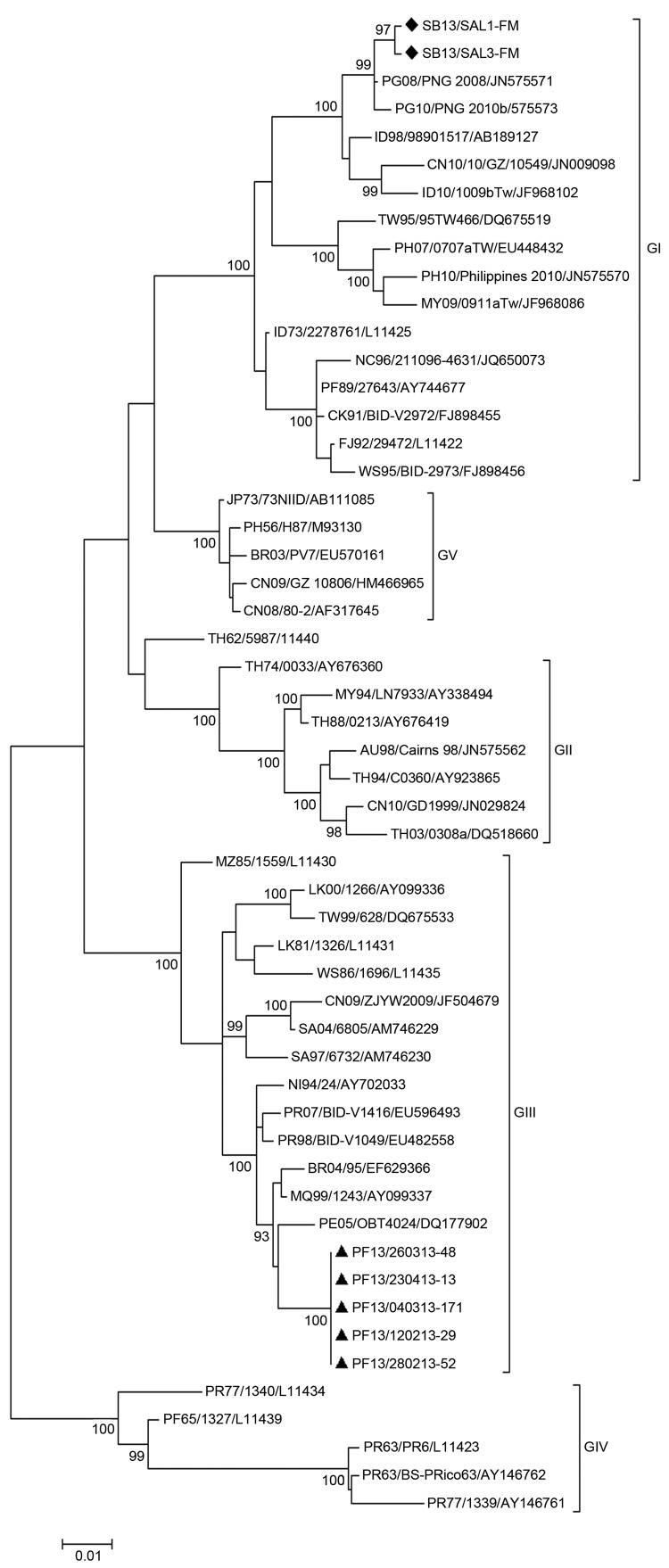
Evolutionary relationships of 54 dengue virus type 3 (DENV-3) gene sequences. The phylogenetic tree was obtained by using the maximum-likelihood method based on the Kimura 2-parameter model and MEGA 5 software (http://www.megasoftware.net/). The percentage of replicate trees in which the associated taxa clustered together in the bootstrap test (1,000 replicates) is shown for values >90. Each strain is labeled by country of origin/strain name/GenBank accession number (if available). Black diamonds indicate DENV-3 strains sequenced in this study that were isolated during the DENV-3 epidemic in the Solomon Islands in 2013. Black triangles indicate DENV-3 strains sequenced in this study that were isolated during the DENV-3 epidemic in French Polynesia in 2013. Genotypes are indicated on the right side of the tree. Scale bar indicates nucleotide substitutions per site.

All sequenced DENV-3 strains isolated in the Solomon Islands belonged to genotype I, and all sequenced DENV-3 strains isolated in French Polynesia belonged to genotype III (Figure 2, Appendix). Phylogenetic analyses suggested that the recent DENV-3 outbreak in the Solomon Islands resulted from introduction of a strain from Indonesia that might have been circulating unrecognized in Papua New Guinea for 4–5 years. Previous reemergence of DENV-4 into the Pacific Islands might have resulted from introduction of a strain from Indonesia into the Solomon Islands (*4**,**5**,**11*).

In contrast, epidemiologic and phylogenetic evidence suggested that the outbreak of DENV-3 infection in French Polynesia resulted from introduction of virus from South America (French Guiana). Before 1990, most dengue outbreaks in the South Pacific region were caused by DENV that originated in Latin America. However, since that time most outbreaks have been caused by DENV from Southeast Asia (*1**–**4**,**12**,**13*). Recent transmission of DENV-3 from South America into French Polynesia is a timely reminder of the ongoing risk for DENV infection from a region in which DENV transmission is becoming hyperendemic. It remains to be seen whether, in the South Pacific, genotypes I and III of DENV-3 will maintain independent areas of transmission, they will co-circulate in the same localities, one will become dominant, or populations of the South Pacific Island nations are large enough for these sorts of dynamics to occur.

## Conclusions

As anticipated by Li et al. (*4*), reemergence of DENV-3 in the South Pacific Islands in 2013 supports the contention that birth and immigration rates (1.2% and 1.1%, respectively, in French Polynesia in 2010) in these island nations provide sufficient susceptible hosts for DENV to cause an epidemic every 4–5 years and for a given DENV serotype to reappear every 12–15 years. Moreover, the finding that recent DENV-3 outbreaks were caused by multiple genotypes supports observations made during outbreaks of DENV-1 infection in 2001–2004 (*2*). These observations also suggest that the principal determinant of DENV reemergence in the South Pacific Islands is herd immunity, rather than the genotype of the DENV being introduced.

Genotype III of DENV-3 is believed to have originated in Sri Lanka, spread to eastern Africa in the mid-1980s, and then to South America in the mid-1990s (*14*). The discovery that DENV-3 genotype III has recently been introduced into the South Pacific Islands from South America indicates that this lineage might be the first DENV lineage to have circumnavigated the globe. Although the reemergence of DENV-3 in the South Pacific Islands in 2013 was anticipated, the reemergence of DENV-1 in New Caledonia in 2012 (*15*) and in French Polynesia in 2013 was not anticipated. Reports of co-circulation of multiple DENV serotypes are becoming more frequent in the South Pacific region and might reflect consequences of incomplete vector control (i.e., sufficient to prevent a major dengue outbreak but also preventing sufficient DENV transmission for development of effective herd immunity). Whether DENV-3 genotype I, genotype III, or both genotypes, will displace DENV-1 is not known.
